# A Facile One-Pot Synthesis of Biomimetic Photocatalyst Zn(II)-Porphyrin-Sensitized 3D TiO_2_ Hollow Nanoboxes and Synergistically Enhanced Visible-Light Degradation

**DOI:** 10.1186/s11671-018-2745-5

**Published:** 2018-10-24

**Authors:** Lianqing Chen, Chengjiang Zhang, Lamei Wu, Kangle Lv, Kejian Deng, Tsunghsueh Wu

**Affiliations:** 1 Key Laboratory of Catalysis and Materials Science of the State Ethnic Affairs Commission & Ministry of Education, South-Central University for Nationalities, Wuhan, 430074 People’s Republic of China; 2 Department of Chemistry, University of Wisconsin-Platteville, Platteville, WI 53818 USA

**Keywords:** Biomimetic photocatalyst, 3D hollow nanoboxes, Singlet oxygen, Visible-light photodegradation, Advanced oxidation processes (AOPs)

## Abstract

A serials of biomimetic photocatalyst zinc(II) *meso*-tetra(4-carboxyphenyl)porphyrinato (ZnTCP)-sensitized 3D hierarchical TiO_2_ hollow nanoboxes (TiO_2_-HNBs) assembled by six ordered nanosheets with dominant {001} facets exposure (ZnTCP@TiO_2_-HNBs) have been successfully synthesized by a facile one-pot solvothermal method *via* a topological transformation process with TiOF_2_ as template. Infrared spectra (IR), UV-vis spectroscopy, and X-ray photoelectron spectroscopy (XPS) confirmed that ZnTCP played a decisive role in constructing 3D hollow nanoboxes through the formation of ester bond combined to TiO_2_-HNBs, which also provided a transferring photo excited electrons bridge to sensitize TiO_2_-HNBs for enhancing visible-light response. Due to the superior sensitization and biomimetic activity of ZnTCP, the photodegradation rate of rhodamine B (RhB) of as-prepared ZnTCP@TiO_2_-HNBs with ZnTCP/TiOF_2_ mass ratio of 2% (T-2p) improves 3.6 times compared to that of TiO_2_-HNBs with a degradation yield of 99% for 2 h under simulated sunlight irradiation (> 420 nm). The enhanced photodegradation ability was attributed to synergistic visible photocatalytic mechanism of biomimetic catalyst, which can not only produce hydroxyl radical (•OH) and superoxide radical (•O_2_^−^) coming from the excitation process of ZnTCP sensitized TiO_2_-HNBs, but also generate singlet oxygen (^1^O_2_) that was only provided by biomimetic enzyme porphyrins. Furthermore, the photocatalyst showed good recycling stability and dispersibility after five rounds, ascribed to ZnTCP strong chemical bonding to the support TiO_2_-HNBs. By means of electrochemical cyclic voltammetry analysis, the effect of central zinc ions and parent porphyrin rings on the redox property of biomimetic catalyst was studied.

## Background

All kinds of organic dyes play a non-substitutable role in various industrial application fields, including dyeing of textiles, leather, or paper [1]. However, their toxicity, diversity, and persistence directly affect the health of ecosystems and pose a direct threat to humans through contaminated drinking water supplies [2]. How to find a simple way to remove synthetic dye pollutants from industrial wastewater effluent is recognized as a pressing challenge.

Numerous methods have been developed to handle the organic dye removal from waste water including physical, biological, and chemical methods [3–5]. The physical ways such as adsorption, coagulation, ion-exchange and membrane filtration are employed to remove organic pollutant [6, 7], whereas physical methods only transfer the organic molecules away from water phase, and it does not fundamentally resolve the problem. In addition, biological processes usually occur under relatively mild conditions, due to their inherent time-consuming procedure and their intolerance to organic pollutants, thus making it difficult for large-scale industrial applications. Because it used the use of solar energy for the decomposition of dye pollutants, the formed AuAgCuBi_2_O_4_ composite exhibits an excellent photocatalytic activity toward the degradation of RhB under simulated-sunlight irradiation. And the composites exhibit a significantly enhanced photocatalytic activity [8–11]. Based on the above considerations, the effective removal of organic pollutants from waste water is far from satisfactory, and there is still an urgent need to develop cost-effective and environment-friendly strategies. Advanced oxidation processes (AOPs) based on the production of active species, such as hydroxyl free radicals, which can handle in the problem of dye destruction in aqueous systems, have been used to no selective oxidation of a wide range of organic pollutants [12]. Some typical AOPs techniques have been successfully developed for the treatment of organic pollutants, including Fenton schemes, ozone systems, semiconductor photocatalysts, and electrochemical catalysis. In recent years, due to its simple operation and environmental compatibility, considerable attention has been devoted to the degradation of organic pollutants catalyzed by biomimetic catalysis, which gathers the advantages of enzyme’s recognition and chemical catalysis [13].

Compared to chemical catalysis, the high selectivity is one of the greatest advantages of biomimetic catalysis, and it can be operated under mild and environmentally friendly conditions, which may significantly reduce energy input and waste generation. Recently, the metalloporphyrin and its derivatives, which can mimic the oxidation characteristic process of peroxidase enzymes or iron-containing oxygenase, have attracted much attention to use as oxidative degradation of persistent organic pollutants due to good chemical stability and their high absorption coefficient within the solar spectrum. Zhao reported iron (II) bipyridine supported on an ion-exchanged resin could effectively activate O_2_ to degrade organic dyes in water under visible-light irradiation [14]. Collins synthesized a series of iron-centered tetraamido macrocyclic ligand complexes, which were able to activate both H_2_O_2_ and O_2_ to degrade organic contaminants in heterogeneous aqueous systems to mimic peroxidase-like processes at enzymatic rates. Our groups reported iron tetra (1, 4-dithiin) porphyrazine supported on anion exchange resin (amberlite CG-400) exhibited excellent oxidative degradation of organic dyes. Taking account into the reported, it is found that these metalloporphyrins and its derivatives are all based on the center iron ion, due to the variable valence of iron ion. However, because Fe^2+^ is much more difficult to be chelated into the porphyrin ring and atmospheric instability, this process is usually performed under harsh conditions and long reaction times.

As we all know, zinc porphyrins and its metalloporphyrin have exhibited attractive properties, such as high stability, good photosensitivity, non-toxic, and facile synthesis, which are favored by more and more scientists. In particular, the photosensitivity of zinc porphyrins can induce singlet oxygen quantum yield to make use for photodynamic therapy and photocatalytic oxidative degradation of organic pollutants [15]. These excellent properties inspire us to find better ways designing facile synthesis zinc porphyrin composites to study its photocatalytic oxidation of organic pollutants. At the same time, considering the hydrophilicity of zinc porphyrin, four carboxylic groups were introduced into its peripheral big ring.

Another urgent problem needed to resolved the supports of zinc porphyrin composites. Corresponding to our group’s previous reports, metalloporphyrins and metalloporphyrazines only show excellent heterogeneous photocatalytic activity when they are supported on anion exchange resin (Amberlite CG-400) or ion-exchanged resin. However, all kinds of resins cannot generate electron and holes pairs and quickly transfer the charges under visible-light irradiation. Therefore, we focus our eyes on a typical semiconductor photocatalyst TiO_2_.

Until now, anatase TiO_2_ has become one of the most important photocatalysis due to its potential application in solving environmental pollution and energy crisis such as photocatalytic degradation of organic pollutants in wastewater due to its nontoxicity, higher chemical stability, and photostability. However, it can only absorb a very small ultraviolet part (about 4.5%) of the solar light due to the relative wide bandgap of anatase TiO_2_ (3.2 eV), which severely restricts its practical application [16–18]. Therefore, it is of great importance issue for enhancing the visible-light photoreaction activity of TiO_2_-based photocatalysts to make full use of the abundant amount of the solar spectrum, but remains a great challenge. Up to date, there has been a significant amount of studies devoted to improve the visible-light utilization of TiO_2_ by semiconductor coupling [19–21], metal and non-metal ions doping [22, 23], and surface sensitized modification [24–26]. This can be explained by the fact that the surface oxygen vacancies act as photoinduced charges acceptors and adsorption sites suppress the recombination of photogenerated charges, leading to an increasing availability of photogenerated electrons and holes for photocatalytic reaction [27–29]. With respect to semiconductor coupling, although it can effectively increase the separation rate of photogenerated electron-hole pairs, their photoreaction efficiencies strongly depend on their junction structures and only obtained too low energy conversion efficiencies for industrial application [30–32]. The doping modification of TiO_2_ is generally rather difficult to preparation with lattice exchange at high temperature and multistep procedures. The surface sensitization approach can dramatically extend the light-responsive region of TiO_2_ from UV to visible-light range. As a good sensitizer, metalloporphyrin and its derivative-sensitized TiO_2_ have attracted a great deal of attention due to their high absorption coefficient within the solar spectra, small singlet-triplet splitting, high quantum yield for intersystem crossing, the long triplet state lifetimes, and good chemical stability [33, 34]. More importantly, porphyrin sensitization is able to extend the bandgap of TiO_2_ optical absorption into the visible-light region, which can efficiently catalyze degradation of organic pollutants. The functional groups around porphyrin ring are the channel making the excited state electrons of the dye transferred to TiO_2_ conduction band [35, 36].

Recently, a great amount of 3D TiO_2_ hollow nanostructures have been synthesized by all sorts of methods, such as 3D hollow nanotubes or microspheres constructed from nanowires, nanosheets, nanorods, or nanoparticles. However, these hollow structures, consisted of randomly assembled building blocks, are mainly based on spherical-like structure. The pursuit of non-spherical-like 3D TiO_2_ hollow nanomaterials, such as box-like or cube-like structure, composed of an ordered hierarchical structure still remains a challenge issue. To date, both experimental and theoretical analysis has demonstrated that the high energy {001} facets of anatase TiO_2_ can efficiently separate photogenerated electron-hole pairs and narrow the bandgap of TiO_2_, which has attracted an explosion of research interest [37]. Based on the above considering of morphology and facet modification, how to construct 3D hierarchical TiO_2_ hollow box-like structures, assembled by six ordered arranged sheets, with dominant exposure of {001} facets to enhance visible-light absorption, remains still a difficult problem. Thus, there is an urgent demand to develop a facile and one-pot topological synthetic strategy to synthesize porphyrin-sensitized 3D hollow TiO_2_ structures to obtain visible-light-driven response. To our best knowledge, the in situ fluoride-induced self-transformation cubic TiOF_2_ to hollow nanoboxes’ preparation of zinc porphyrin-sensitized TiO_2_ composite photocatalyst by solvothermal treatment has not been reported.

Herein, we proposed *meso*-tetra (4-carboxyphenyl) porphyrinato zinc(II) (ZnTCP) with carboxyl group onto porphyrin periphery as a sensitizer and employed a facile one-pot topological solvothermal method to prepared ZnTCP-sensitized TiO_2_ hollow nanoboxes (ZnTCP@TiO_2_-HNBs) with dominant {001} facets exposure through the formation of steady covalent bonds between TiO_2_-HNBs and zinc porphyrins. We systematically studied the effect of zinc porphyrin mass content on the structure and visible photocatalytic activity of zinc porphyrin modified TiO_2_ hollow nanoboxes (ZnTCP@TiO_2_-HNBs).

## Methods

### Materials

All reagents and solvents were of reagent grade quality obtained from commercial suppliers (Wuhan Guoyao Chemical Reagent Co. Ltd.) and used directly without further purification. All the solutions were prepared with distilled water.

### Synthesis of ZnTCP-Sensitized TiO_2_-HNBs (ZnTCP@TiO_2_-HNBs)

#### TiOF_2_ Precursor was Prepared as the Nanocubic Template Firstly

In a typical procedure, 30 mL acetate, 5 mL hydrofluoric, and 15 mL tetrabutyl titanate were added dropwise into 100 mL polyvinyl fluoride beaker and continuously stirred for 25 min. Then, the mixture was transferred into a dried 100-mL Teflon-lined autoclave and kept at 200 °C for 12 h. After being cooled to room temperature, the white powder was washed several times with ethanol and dried at 60 °C under vacuum overnight, then obtains TiOF_2_ nanocubic precursor.

#### Preparation of Zn Porphyrins (ZnTCP)

The preparation procedure included two steps. The first step is synthesis of porphyrin (TCP): typically, 100-mL propionic acid and 4.341 g (28.8 mmol) 4-carboxybenzaldehyde were added into 500-mL three-necked flask, which heated in oil-bath and stirred for 30 min to ensure complete dispersion. A solution of 10-mL propionic acid and 2-mL pyrrole was slowly added dropwise into the above system and refluxed at 140 °C for 2 h. The mixture was cooling down to room temperature and washed with 150-mL hot water twice. Then, using sodium carbonate and sulfuric acid to remove tar and adjust pH value in 5 to 7 respectively. The product was extracted with butanol for three times; finally, the product was concentrated and dried by rotary evaporation. ^1^H NMR spectrum of TCP showed the expected signals such as -OH, Ar-H (phenyl), and -CH (pyrrole) protons at 4.12, 6.98–7.02, and 7.46–7.57 ppm, respectively. Inner core NH protons appear as a deuterium exchangeable broad chemical shift at = − 3.34 ppm as a consequence of the 18 π-electron systems of the planar structure. IR (KBr, cm^−1^) spectra, 3363 for -OH, 2927, 2852 for C-H, 1712 (C=O), 1623, 1488, and 1376 for C=C, 1053 for COOH, confirms the TCP structure. The mass spectrum exhibited a molecular ion peak at *m/z* = 614.14 [M + 1]^+^. UV-vis [DMF, max/nm] B-band at 419 nm and Q-band at 553 and 598 nm.

**Scheme 1:**
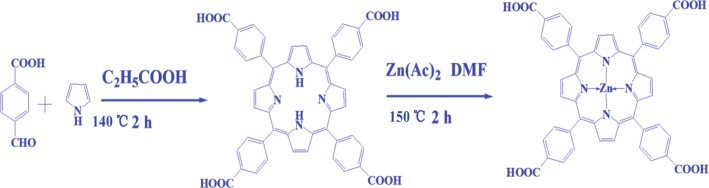
Schematic illustration of synthesis of ZnTCP complex

Secondly, synthesis of ZnTCP was as follows: 0.544 g porphyrins and 0.20 g zinc acetate were dissolved in DMF solvent. The mixture solution was heated and refluxed at 150 °C for 2 h using oil bath with continuous stirring. Then cooling it down to room temperature, the product was concentrated and added 100-mL deionized water after purple was precipitation completely. Finally, the water was removed by rotary evaporation and *meso*-tetra (4-carboxyphenyl) porphyrin zinc (II) (ZnTCP) was obtained. In the ^1^H NMR spectra of ZnTCP, inner core NH protons disappeared; other protons showed the similar chemical shift at 4.12, 6.98–7.02, and 7.46–7.57 ppm. From IR spectrum, the sharp peak of C=O bond vibration around 1720 cm^−1^ had been enhanced after the cyclotetramerization reaction, 3352 cm^−1^ for -OH, 2928, 2853 cm^−1^ for C-H, 1619, 1504 cm^−1^ and 1378 cm^− 1^ for C=C, 1231 and 1017 cm^−1^ for C=O, confirmed the structure of ZnTCP. The mass spectrum data of ZnTCP contained a strong peak at m/z = 678.11 [M + 1]^+^ for the parent ring ion. The XPS spectrum proved the presence of 2p1/2 Zn(II) peaks with binding energy of 1021.5 eV and 2p3/2 with binding energy of 1045.6 eV. UV-vis [DMF, max/nm] B-band at 432 nm and Q-band at 561 and 600 nm. The synthetic procedure of ZnTCP is illustrated in Scheme 1.

#### A Facile One-Pot Synthesized of ZnTCP-Sensitized TiO_2_-HNBs (ZnTCP@TiO_2_-HNBs)

**Scheme 2:**
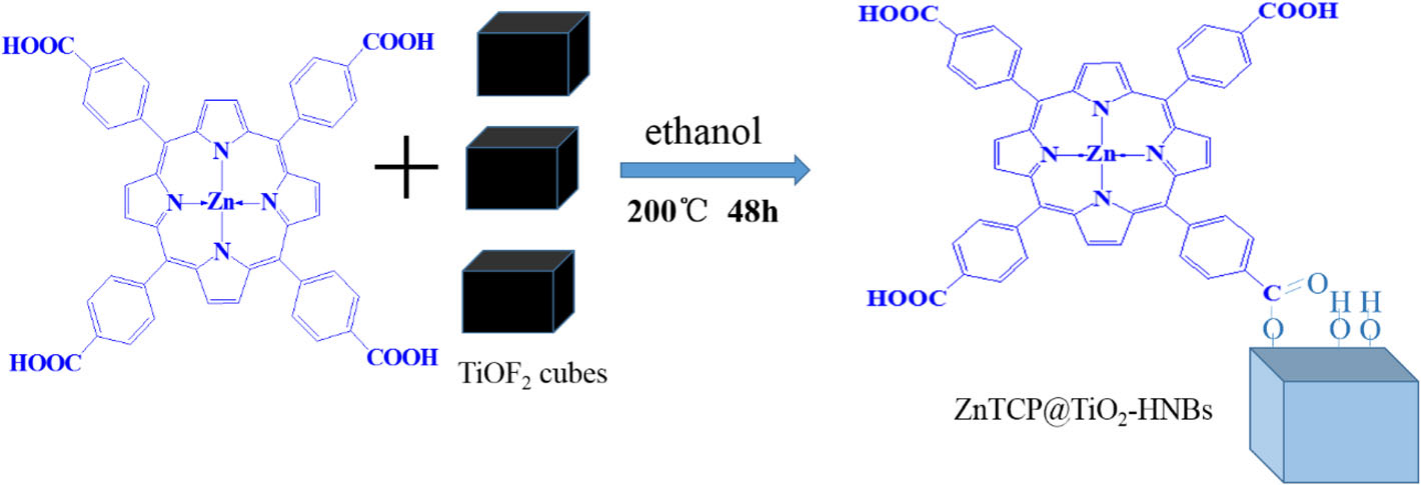
Schematic illustration of preparation of ZnTCP@TiO_2_-HNBs

Using TiOF_2_ as the template, ethanol as solvent and zinc porphyrins as a sensitizer and TiO_2_ hollow nanoboxes as supports, porphyrins sensitized 3D hierarchical titanium dioxide hollow nanoboxes with exposed high energy {001} facets were prepared via a topological transformation process. In a typical procedure, 300 mg TiOF_2_, 70 mL ethanol, and 3 mg ZnTCP were placed in Teflon-lined autoclave and under reaction for 48 h at 200 °C, then cooling it down to room temperature; the product was concentrated by rotary evaporation and dried at 60 °C under vacuum overnight. Then, obtained product was denoted as T-1p, where 1 represents ZnTCP/TiOF_2_ mass ratio. For comparison, different samples were also synthesized under other identical conditions except the amount of zinc porphyrins. They are denoted as T-0p, T-1p, T-2p, T-3p, and T-5p, respectively. The one-pot synthetic process of ZnTCP@TiO_2_-HNBs is shown in Scheme 2.

### Characterization

Fourier transform infrared spectroscopy (FT-IR) was measured on a NeXUS 470 spectrometer using KBr pellet technique. The crystalline structure of these catalysts was characterized by powder X-ray diffraction (XRD) employing a scanning rate of 0.05°s^−1^ in a 2*θ* range from 10 to 80°, in a Bruker D8 Advance using monochromatized Cu-Ka radiation. X-ray photoelectron spectroscopy (XPS) was recorded on a VG Multilab 2000 photoelectron spectrometer using monochromatic Mg K*a* radiation under vacuum at 2 × 10^−6^ Pa. All the binding energies were referenced to the C1s peak at 284.8 eV of the surface adventitious carbon. The morphologies and microstructures of as-prepared samples were analyzed by a field emission scanning electron microscope (SEM) (Hitachi, Japan) and a transmission electron microscope (TEM) (Tecnai G^2^ 20, USA). The UV-vis diffuse reflectance spectroscopy (DRS) were collected using a Shimadzu UV-2600 spectrophotometer from 240 to 800 nm using BaSO_4_ as background. Photoluminescence (PL) spectra were obtained on a Hitachi F-7000 Fluorescence Spectrophotometer. The cyclic voltammetry (CV) measurements were performed on electrochemistry workstation controlled by an external PC and utilizing a three-electrode configuration at room temperature.

### Photocatalytic Measurements

All photoreaction experiments were carried out in a self-made photocatalytic reactor system, the photocatalytic activities of as-prepared samples were evaluated by organic dyes RhB (1 × 10^−5^ mol/L) as the target pollutant under visible-light irradiation. The specific process is as follows: 50-mg photocatalyst and 50-mL initial concentration of 5 × 10^−4^ mol/L RhB solution were added into a cylindrical reaction vessel by ultrasound dispersion with a 14 cm^2^ plane side and 7 cm height, and then the mixed solution was oscillated in the dark overnight. After reaching adsorption equilibrium, the photocatalytic reaction was initiated by irradiating the system with a 210 W xenon lamp with light filter (*λ* > 420 nm) to ensure illumination by visible-light only, the system was continuously cooled by water, which were used to maintain the system at room temperature. At given time intervals, 3.5 mL aliquots were collected and centrifuged and then remove the catalyst particles for analysis. The concentration of RhB at different intervals was monitored by UV-vis spectroscopy. All of the measurements were carried out at room temperature.

The photoluminescence (PL) technique was used to study the active species produced during catalyst degradation. According to the precious reported [38], coumarin, 4-chloro-7-nitrobenz-2-oxa-1, 3-diazole (NBD-Cl), and 1, 3-Diphenylisobenzofuran (DPBF) were used as fluorescent probe to detect hydroxyl radical (•OH), superoxide radical (•O_2_^−^), and singlet oxygen (^1^O_2_), respectively. ZnTCP@TiO_2_-HNBs catalyst and water react to form the active species hydroxyl radicals (•OH) and then are rapidly trapped by coumarin to generate 7-hydroxycoumarin which has strong fluorescence property. Typically, the detected procedure is as follows: The suspensions of catalysts (1.0 g/L) containing coumarin (0.1 mmol/L) is mixed under magnetic stirring and then was shaken overnight. The mixture was irradiated with 210 W xenon lamp light and taken the sample at the set 2 min intervals (or 4 min or 15 s). The fluorescence spectrophotometer was used to analyze filtrate by the excitation with the relevant wavelength.

## Results and Discussion

### TEM and SEM Images

**Fig. 1:**
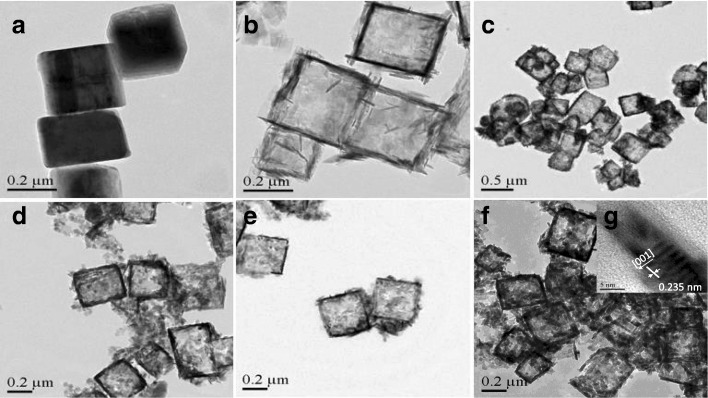
TEM images of as-synthesized catalysts: **a** TiOF_2_, **b** TiO_2_-HNBs, **c** T-1p, **d** T-2p, **e** T-3p, and **f**, **g** T-5p

The morphology and crystal planes of TiOF_2_, TiO_2_-HNBs, and ZnTCP@TiO_2_-HNBs samples were observed by TEM illustrated in Fig. 1. Figure 1a shows that TiOF_2_ reveals uniform solid cubic shape and smooth surface with an average size of 250 nm. As shown in Fig. 1b, it can be seen that the morphology of TiO_2_-HNBs transformed to uniform hollow box-like shape composed of six ordered faces with average side length of 260 nm, which was consistent with the cubic TiOF_2_ template. Seeing carefully from Fig. 1c–f, it can be found that 3D hollow structures of ZnTCP@TiO_2_-HNBs still displayed well-defined box-shaped morphology during the fluoride-induced self-transformation cubic TiOF_2_ to ZnTCP-sensitized TiO_2_ hollow nanoboxes process, which were well-shaped with some hairy tentacles of ZnTCP covered on the surface of hollow boxes [39]. HRTEM images (see the insert of Fig. 1g) were obtained to confirm the lattice fringes were about 0.235 nm, which was good agreement with the high energy {001} crystallite facet of anatase TiO_2_. The surface of ZnTCP@TiO_2_-HNBs lattice diagram exhibited 3D hierarchical hollow nanoboxes consisting of six ordered dominant exposed {001} facets nanosheets.

**Fig. 2:**
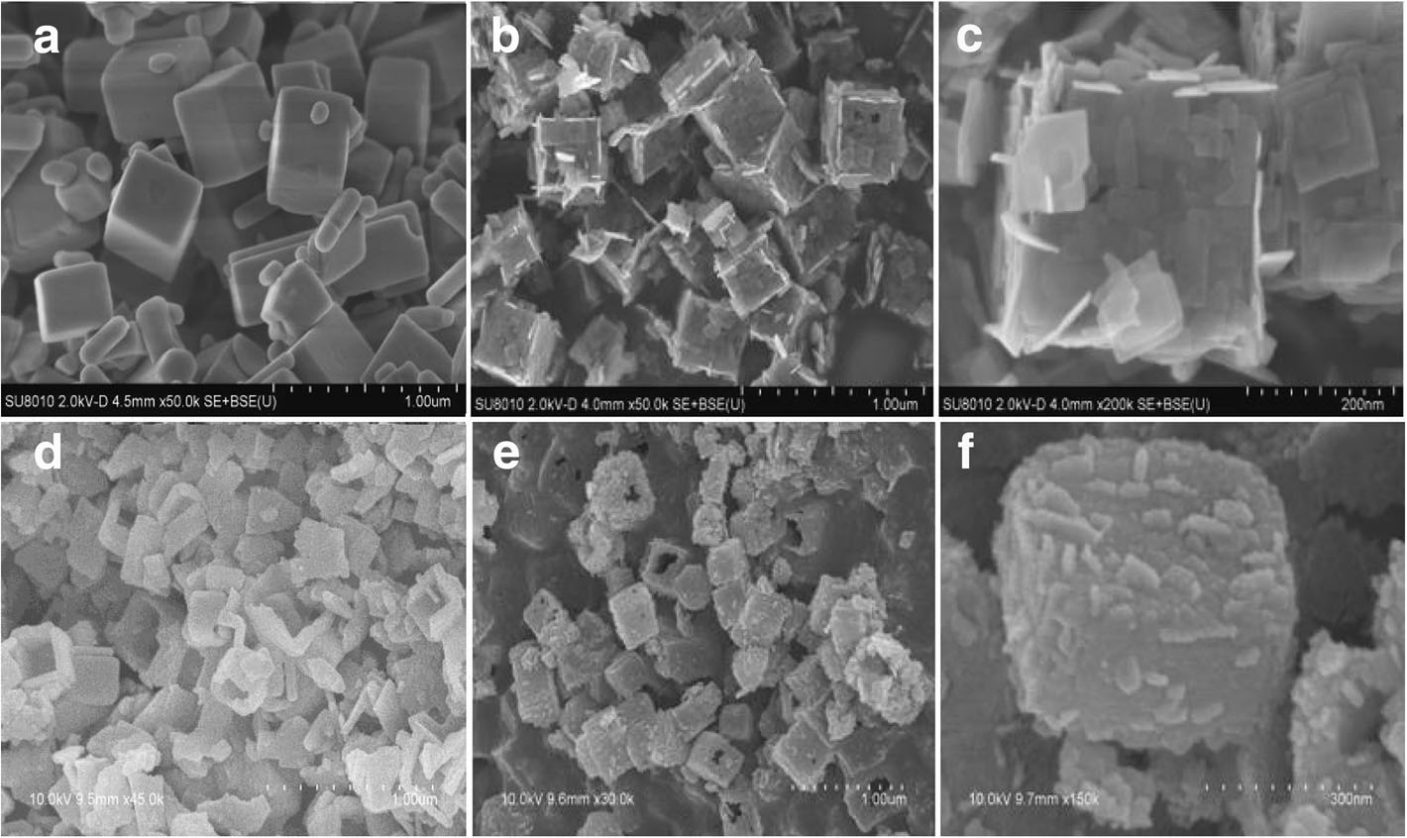
SEM images of as-prepared catalysts: **a** TiOF_2_, **b** TiO_2_-HNBs, **c** T-1p, **d** T-2p, **e** T-3p, and **f** T-5p

The surface morphology of TiOF_2_, TiO_2_-HNBs, and ZnTCP@TiO_2_-HNBs was identified from the SEM images illustrated in Fig. 2. Figure 2a is made up of uniform and completely cubic TiOF_2_ in accord with the TEM images. As displayed in Fig. 2b, the images of TiO_2_-HNBs showed well-shaped hollow boxes morphology, which derived from the template TiOF_2_ cubes in the topological transformation procedure and enclosed by six ordered TiO_2_ nanosheets arrays. As displayed in Fig. 2c–f, all ZnTCP@TiO_2_-HNBs samples also exhibited completely hollow box-like structures with hairy tentacles of Zn(II) porphyrin covering on the surface of nanoboxes, which was well consistent with TEM results, implying the successful one-pot topological transformation process from cubic TiOF_2_ to TiO_2_ hollow nanoboxes with ZnTCP in situ participation.

**Fig. 3:**
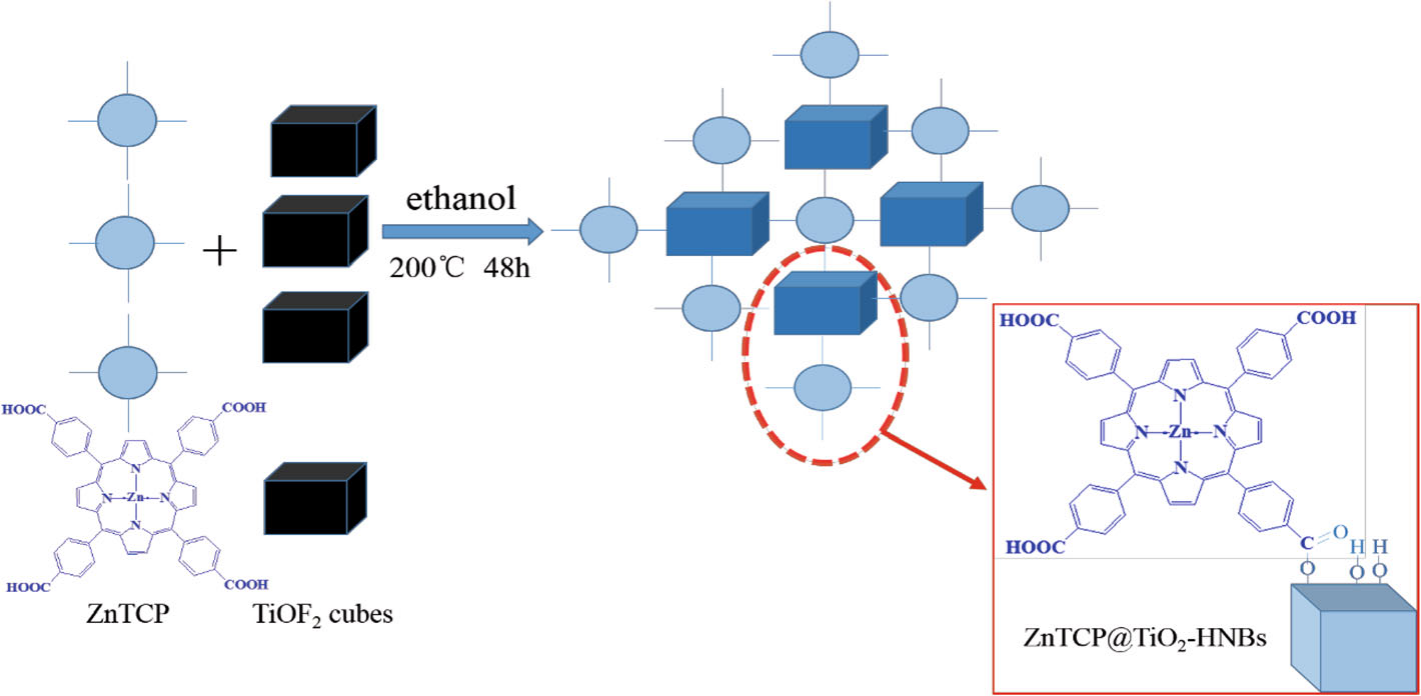
The proposed formation process of Zn(II) porphyrin sensitized TiO_2_-HNBs (ZnTCP@TiO_2_-HNBs)

As described in Fig. 3, the probable formation mechanism of Zn(II)-porphyrin-sensitized 3D TiO_2_hollow nanoboxes (ZnTCP@TiO_2_-HNBs) has been proposed via a process of topological transformation involving template participation. Ethanol provided mild reaction conditions by using a solvent, whose dehydration reaction (Eq. 1, main) and ester reaction between ZnTCP and ethanol (Eq. 2) supplied H_2_O and promoted the hydrolysis of cubic TiOF_2_, which can topologically transform to anatase TiO_2_ hollow box-shape nanocrystals during solvothermal reaction (Eq. 3). Because the adsorption of F^−^ ion on the surface of TiO_2_ nanocrystals can sharply reduce the surface energy of (001) facets, fluoride ions facilitate the formation of high-energy anatase TiO_2_ nanosheets and it is understandable that precursor TiOF_2_ can transform into TiO_2_ hollow nanoboxes assembly by six ordered nanosheets with exposed high-energy (001) facets. The peripheral tetracarboxyl group of ZnTCP formed the strong ester bond combined to the support TiO_2_-HNBs (Eq. 4).

1 $$ {\displaystyle \begin{array}{l}{\mathrm{TiO}\mathrm{F}}_2+2{\mathrm{CH}}_3{\mathrm{CH}}_2\mathrm{O}\mathrm{H}\circledR {\mathrm{TiO}}_2+2\mathrm{HF}+2\mathrm{CH}={\mathrm{CH}}_2\left({\mathrm{CH}}_3{\mathrm{CH}}_2{\mathrm{OCH}}_2{\mathrm{CH}}_3\right)\\ {}\mathrm{alcohol}\;\overset{\mathrm{dehydration}}{\to}\;\mathrm{alkene}\ \left(\mathrm{ester}\right)+{\mathrm{H}}_2\mathrm{O}\end{array}} $$

2 $$ \mathrm{Zn}\left(\mathrm{II}\right)\hbox{-} \mathrm{porphyrin}\hbox{-} \mathrm{COOH}+{\mathrm{CH}}_3{\mathrm{CH}}_2\mathrm{O}\mathrm{H}\circledR \mathrm{Zn}\left(\mathrm{II}\right)\hbox{-} \mathrm{porphyrin}\hbox{-} {\mathrm{COOCH}}_2{\mathrm{CH}}_3+{\mathrm{H}}_2\mathrm{O} $$

3 $$ {\mathrm{TiO}\mathrm{F}}_2+{\mathrm{H}}_2\mathrm{O}={\mathrm{TiO}}_2\left(\mathrm{anatase}\ \mathrm{crystal}\right)+2\mathrm{HF}\left(\mathrm{in}\ \mathrm{situtransformation}\right) $$

4 $$ \mathrm{Zn}\left(\mathrm{II}\right)\hbox{-} \mathrm{porphyrin}\hbox{-} \mathrm{CO}\mathrm{OH}+{\mathrm{TiO}}_2\hbox{-} \mathrm{O}\mathrm{H}=\mathrm{Zn}\left(\mathrm{II}\right)\hbox{-} \mathrm{porphyrin}\hbox{-} \mathrm{CO}\hbox{-} \mathrm{O}\hbox{-} {\mathrm{TiO}}_2 $$

### XRD Analysis

**Fig. 4:**
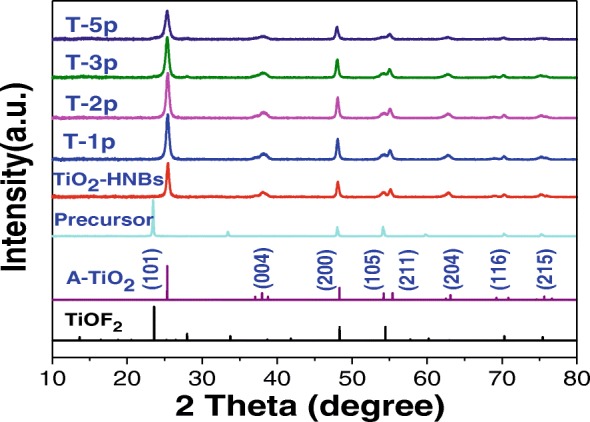
XRD patterns of as-prepared samples

It is well accepted that the crystal planes of TiO_2_-based catalysts play a very important role for their photocatalytic properties. Figure 4 displays the XRD pattern analysis of all as-prepared samples. Looking at the curve relative to TiOF_2_ precursor, {100}, {200}, and {210} crystal planes of TiOF_2_ corresponding to 2*θ* = 24.05°, 48.34°, and 54.45° were clearly observed. For all TiO_2_ samples, a broad peak at 2*θ* = 25.37° was observed, corresponding to the {101} plane diffraction of anatase TiO_2_ (JCPDS No. 21–1272), different from 24.05°, which is attributed to the diffraction peak of TiOF_2_, indicated that all samples successfully completed the topotactic transformation process with TiOF_2_ as template [40]. Due to F^−^ ions as shape control agent, there was no rutile TiO_2_ patterns formed during the synthesized process. Compared toTiO_2_-HNBs (T0p), the diffraction peaks about {101} and {200} crystal face of Zn(II) porphyrin-sensitized TiO_2_-HNBs samples become sharper, implied higher in the degree of crystallization, which is ascribed to the fact that the increase of Zn(II) porphyrin amount led to the concentration increase of ester chemical bonds between the TiO_2_ surface hydroxyl group and peripheral tetracarboxyl group of ZnTCP. The other characteristic diffraction peaks of TiO_2_ samples in XRD patterns did not exhibit any shifts or any changes in peak shape, which indicated that ZnTCP modification and sensitization did not change crystal structure of TiO_2_-HNBs.

To further investigate the crystallite sizes of ZnTCP@TiO_2_-HNBs samples, Scherrer equation was used based on their {101} diffraction peak [41, 42]. As described in Table 1, all the samples indicated their average particle sizes at about 260 nm according to structural analysis, in accord well with the SEM and TEM measurements. However, the “*d*” space value of ZnTCP@TiO_2_-HNBs samples did not increase compared with TiO_2_-HNBs, implying the unit cell dimension of as-prepared ZnTCP@TiO_2_-HNBs catalyst was not likely to change during Zn(II) porphyrin-sensitized procedure.

**Table 1:** Structural analysis of various samples

Samples	XRD_(101)_ relative intensity (%)	XRD_(101)_ peak position (°)	Crystal size^a^ (nm)	*d*-spacing (Å)
TiO_2_-HNBs	78	25.41	271	3.5
T-1p	89	25.38	269	3.5
T-2p	100	25.36	262	3.5
T-3p	87	25.37	260	3.5
T-5p	83	25.35	256	3.5

^a^The crystal sizes were obtained based on the {101} diffraction peak by Scherrer equation: $\mathrm{alcohol}\ \overset{\mathrm{dehydration}}{\to }\ \mathrm{alkene}\ \left(\mathrm{ester}\right)+{\mathrm{H}}_2\mathrm{O}$, where *K* = 0.89 stands for the shape factor, *β* is the full-width half-maximum of the diffraction peak, *λ* represents the wavelength of X-ray irradiation (Cu Kα = 0.15418 nm), and *θ* is the X-ray diffraction angle

### UV-vis Spectroscopy

**Fig. 5:**
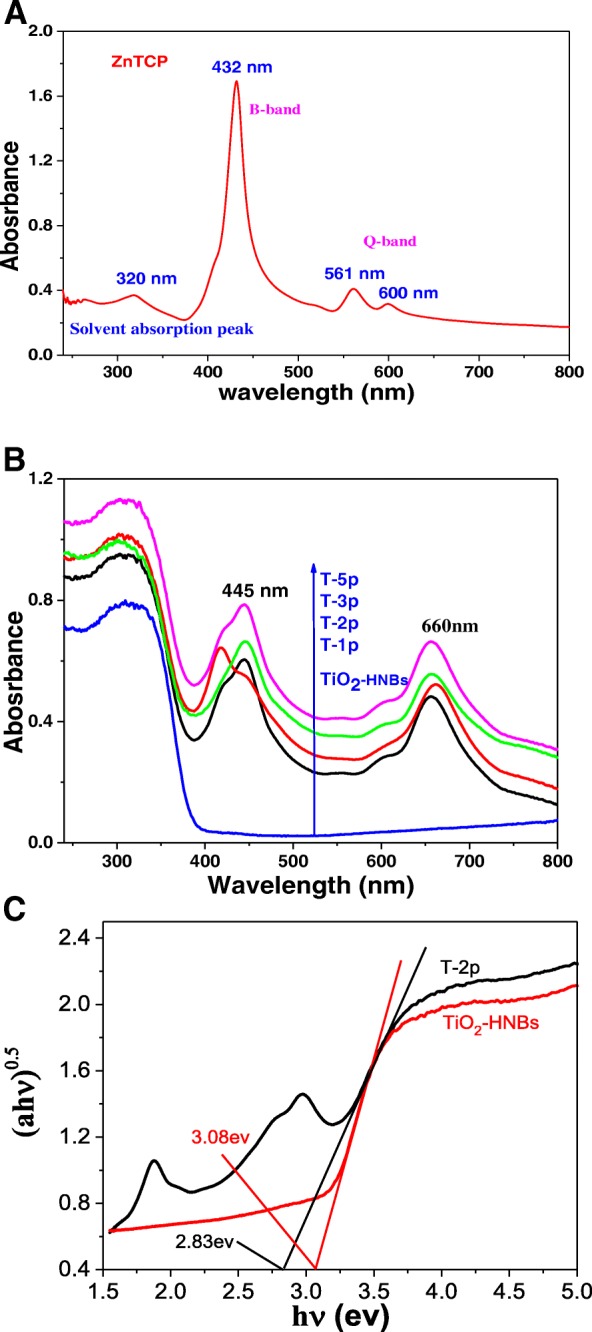
**a** UV-Vis spectra of ZnTCP in 5×10^−4^ mol/L DMF solution. **b** UV-vis diffuse reflectance spectra (DRS) of all the samples. (**c**) Estimated optical absorption edges for the T-2p and TiO2-HNBs

Figure 5 shows the solution UV-vis spectrum of ZnTCP in 5 × 10^−4^ mol/L DMF solution (a) and the UV-vis diffuse reflectance spectroscopy (DRS) of ZnTCP@TiO_2_-HNBs samples and BaSO_4_ was used as the reference sample (b). As Fig. 5a displayed, the strong 432 nm absorption peak was ascribed to the characteristic B-band absorption of porphyrin rings, whereas weaker 561 nm and 600 nm peaks in the visible region were attributed to the characteristic Q-band absorption of ZnTCP, implying the successful synthesis of porphyrin ring. Compared to Fig. 5a, the Q-band absorption peaks of ZnTCP was red-shifted to 660 nm into the longer wavelength visible region after sensitization to TiO_2_. Because there is a presented strong interaction force between zinc porphyrins and TiO_2_-HNBs after hydrothermal reaction, which provides favorable prerequisites for the degradation of organic dyes under the visible-light irradiation. It can be found that TiO_2_-HNBs displayed less absorption above its fundamental absorption edge (> 400 nm), while Zn(II) porphyrin-sensitized TiO_2_ samples displayed an obvious broad visible-light absorbance, as described in Fig. 5b, and the absorption intensities of ZnTCP@TiO_2_-HNBs samples were gradually enhancing much stronger as Zn(II) porphyrin amount increased. In addition, the color of as-prepared samples become deepened gradually from white to blue purple with the increasing of mass ratio of ZnTCP/TiO_2_-HNBs, and it was widely accepted that the purple coloration cause Zn(II) porphyrin-sensitized samples resulted into enhanced visible-light absorption. To further investigate the bandgap in each sample, the energy gap (eV) threshold was obtained using the transversal method, and the graph of the transformed Kubelka-Munk function against the photon energy for samples T-2p and TiO_2_-HNBs are displayed in Fig. 5c. According to the transformed Kubelka-Munk function plot, the energy gaps of *E*_*g*(T-2p)_ and *E*_*g*(TiO2-HNBs)_ are 2.83 and 3.08 eV, respectively. The reduced bandgap arises because ZnTCP can form a local energy state between the valence and conduction bands as ZnTCP concentration increases, which reduce the forbidden band width and the electron transition energy. Meanwhile, we find that the ZnTCP self-doped samples also exhibit very strong UV absorption, which indicates ZnTCP self-doping not only enhances the visible-light response of the catalysts, but also improves their UV light absorption. Thus, it can be predicted that more photo-generated electrons and holes can be excited and can participate in photocatalytic reactions, which is attributed to the increased probabilities of activation by UV and visible light.

### FT-IR Spectra Analysis

**Fig. 6:**
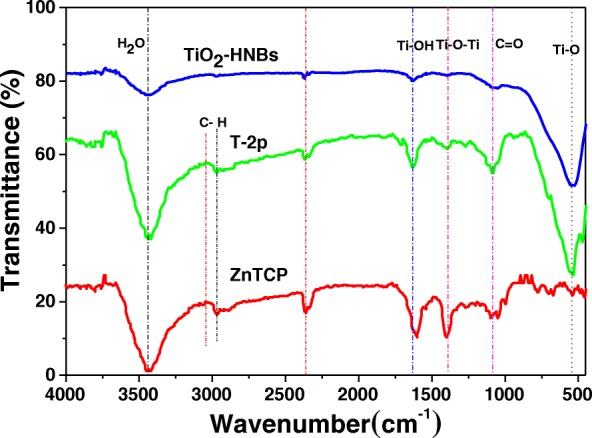
FT-IR spectra of TiO_2_-HNBs, ZnTCP, and T-2p sample

FT-IR spectra analysis is a powerful tool to identify characteristic functional groups [43]. Figure 6 displays the FT-IR spectra of ZnTCP, TiO_2_-HNBs, and T-2p. Looking at the spectra relative to ZnTCP, the broad band at 3443 cm^−1^ is assigned to the stretching vibration of the -OH in the peripheral t carboxyl groups, the characteristic absorption peak of C=O at 1720 cm^−1^. The stretching vibration of Ar-H bond is clearly observed at 2995 cm^−1^. Furthermore, the spectra of ZnTCP show the characteristic absorption peak of benzene ring at 1620 and 1469 cm^−1^ relative to ZnTCP [44].

Seeing from the spectra relative to TiO_2_-HNBs, the stretching vibration and bending vibration of the absorption H_2_O and Ti–OH group on the surface of samples were found about 3446 cm^−1^ and 1627 cm^−1^, respectively. The stretching vibration of Ti-O-Ti is observed at 1388 cm^−1^, and the adsorption peaks around 521 cm^−1^ is assigned to the bending vibrations of Ti-O bond.

Compared to ZnTCP and TiO_2_-HNBs, the T-2p spectra features the characteristic stretching/bending vibration of the Ar-H bond at 2996 cm^−1^ and 1386 cm^−1^ attributed to Ti-O-Ti bond. Furthermore, the spectra of T-2p shows the characteristic absorption peak of phenyl ring at 1621 and 1468 cm^− 1^ corresponding to ZnTCP, indicating the existence of ZnTCP on the surface of sensitized TiO_2_ [44]. However, the signal corresponding to -OH vibration peak disappeared, which demonstrates the formation of ester chemical bond between ZnTCP and TiO_2_. The stretching vibration of the C=O bond of ester group is clearly observed at 1713 cm^−1^ [45], further confirmed ZnTCP combined to TiO_2_-HNBs composites. The above information indicates that the TiO_2_-HNBs surface has been successfully functionalized with ZnTCP molecules. After sensitization, the spectra of T-2p show an increased intensity of the stretching vibration peak at 520 cm^−1^ and the characteristic absorption bands in the region of 1200~1060 cm^−1^ attributed to the CO-O-Ti bond resulting from the esterification between COOH of ZnTCP and OH of TiO_2_ [46]. This reveals that the strong interaction (conjugated chemical bonds) between ZnTCP and TiO_2_-HNBs was established rather than simple physical adsorption. The results displayed on FT-IR spectra were in accordance with the photocatalytic performance of these catalysts, suggesting that the strong conjugated chemical bonds between ZnTCP and TiO_2_ greatly contribute to the improvement of the photocatalytic activity as a transferring electrons bridge.

### XPS Analysis

**Fig. 7:**
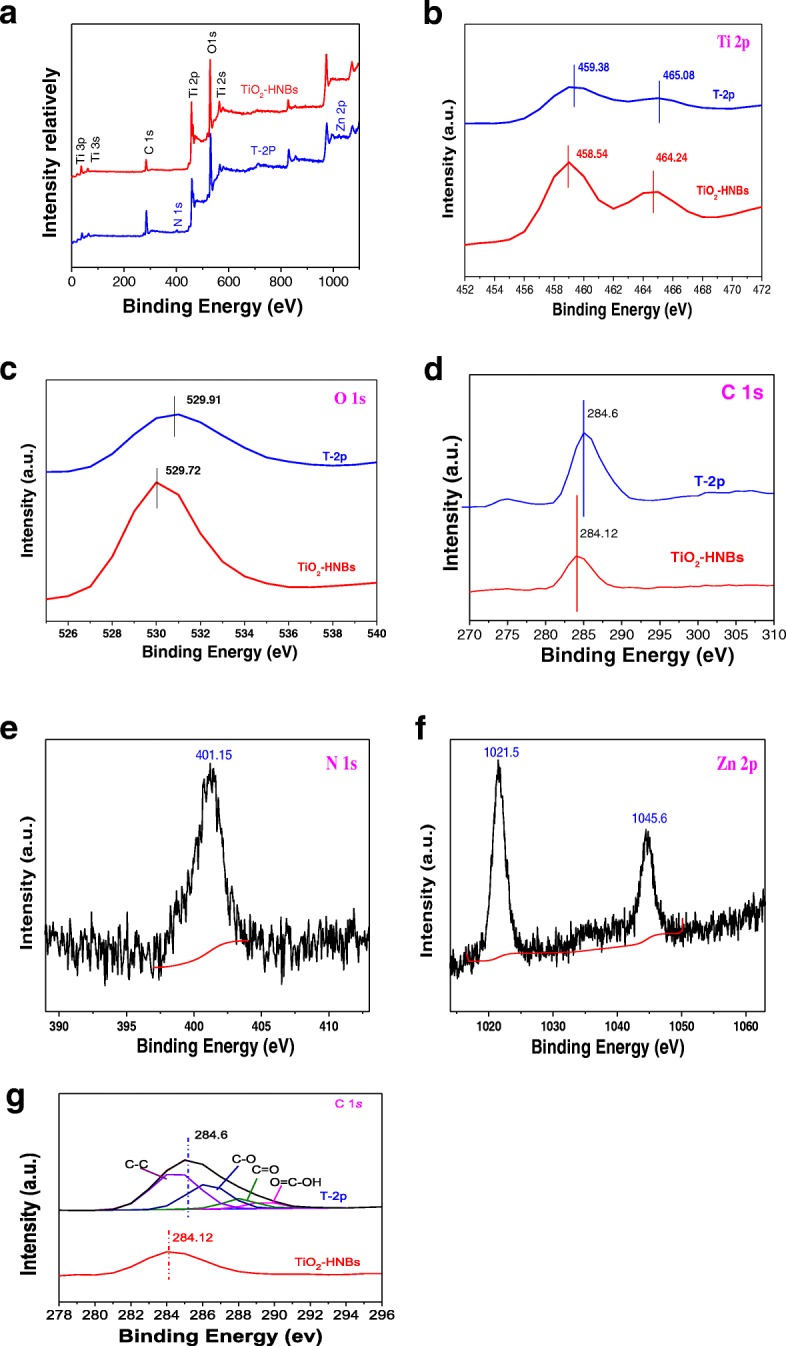
**a** The full spectra of all kinds of elements in sample TiO_2_-HNBs and T-2p; **b** the high-resolution Ti 2p XPS signals of TiO_2_-HNBs and T-2p; **c** the O1s XPS signals of TiO_2_-HNBs and T-2p; **d** the C1s O1s XPS signals of TiO_2_-HNBs and T-2p; **e** the N1s XPS signals of T-2p; **f** the Zn 2p XPS signals of T-2p; **g** the C XPS-peak-differentation-imitating analysis

To further investigate chemical environments of as-prepared catalysts, the X-ray photoelectron spectra (XPS) measurements of TiO_2_-HNBs and T-2p were carried out [43]. As shown in full spectra of Fig. 7a, two elements including Ti and O were both observed at the corresponding positions, respectively. Figure 7b–f displayed the high-resolution XPS spectra of the corresponding element of two samples.

The high-resolution Ti 2p XPS signals of TiO_2_-HNBs in Fig. 7b consisted of two binding energy levels of Ti 2p_1/2_ and 2p_3/2_ at 464.24 and 458.54 eV with a separation energy of about 5.70 eV. However, the binding energy levels of Ti 2p_1/2_ and 2p_3/2_ XPS peaks of T-2p exhibited at 465.08 and 459.38 eV, which shift 0.84 eV toward the lower energy region compared to TiO_2_, indicative of a partial charge transferred from the surface C=O ester moiety to Ti^4+^ centers, which was probably due to the strong interactions between ZnTCP molecules and TiO_2_.

As shown in Fig. 7c, the O1s XPS signals of TiO_2_-HNBs and T-2p displayed at 529.72 and 529.91 eV respectively, with a deviation energy of nearly 0.19 eV. The shifting of lattice oxygen peaks in T-2p toward lower energy, compared to TiO_2_ standard, further verified the different chemical environment due to ZnTCP sensitization. As displayed in Fig. 7d, the C 1s of T-2p appeared the distinct peaks at 284.6 eV, which were ascribed to C=O (and COO) bonds in the peripheral tetracarboxyl group, further confirmed that ZnTCP was well bonded on the surface of TiO_2_. It was found that the weak peak at 401.15 eV of T-2p in Fig. 7e was attributed to N1s of the N=N- bond in the porphyrin ring. The Zn 2p binding energy signals of T-2p appeared at 1021.5 eV and 1044.46 eV in Fig. 7f, which are basically consistent with the complex molecular structure of ZnTCP, further implying it well impregnated onto the surface of TiO_2_. Figure 7g shows the XPS spectra of C 1*s* region for samples. In Fig. 7g, the peak with a binding energy of 284.6 eV can be attributed to the C–C bonds, while the deconvoluted peaks centered at the binding energies of 286.5, 288.3, and 289.6 eV can be assigned to the C–O, C=O, and O=C–OH functional groups, respectively. C 1*s* spectra for the ZnTCP@TiO_2_-HNBs composite are shown in Fig. 7. In the spectrum of ZnTCP@TiO_2_-HNBs, all peaks from oxygen-containing functional groups decreased dramatically in intensity or even disappeared entirely, indicating a significant reduction of GO by solvothermal treatment. In addition, an additional shoulder peak was found, which was usually assigned to the formation of a chemical bond between a carbon atom and a titanium atom in the lattice of TiO_2_-HNBs, which resulted information of Ti–O–C bonds.

### Photocatalytic Activity of ZnTCP@TiO_2_-HNBs Catalysts

**Fig. 8:**
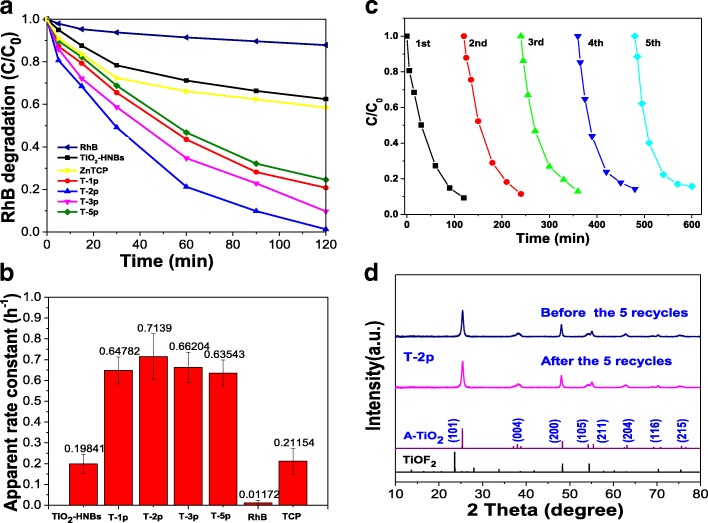
**a** The C _t_/C_0_ vs. time curves of RhB photodegradation under visible-light irradiation. **b** The apparent first-order rate constant of RhB photodegradation. **c** Cyclic degradation curve of T-2p. **d** The XRD before and after recycles of T-2p

The photocatalytic activity of ZnTCP@TiO_2_-HNBs was evaluated by measuring the degradation with RhB as a probe molecule. Figure 8a exhibits the degradation curve of RhB under simulated sun light conditions with as-prepared ZnTCP@TiO_2_-HNBs catalysts. It is clearly observed that, after the adsorption-desorption equilibrium process, the self-degradation of probe molecule RhB can be negligible and the visible-light degradation activities of ZnTCP-sensitized TiO_2_-HNBs samples indicates much higher photocatalytic efficiencies than TiO_2_-HNBs and ZnTCP under visible-light irradiation (λ ≥ 420 nm), which depends on the concentration of RhB versus visible-light irradiation time. The T-2p sample shows highest photocatalytic activity with RhB degradation rate of 99% after 2 h under visible-light irradiation, resulting from its enhanced visible-light response. However, the TiO_2_-HNBs (T-0p) shows little decomposition rate of RhB under the same reaction conditions. In this process, the order of degradation rate is T-2p>T-3p>T-1p>T-5p>ZnTCP>TiO_2_-HNBs.

It is well accepted that the slopes for the curve of RhB intensity versus illumination time (rate constant) represent the photodegradation activity of the samples. Therefore, the exponential curve of the degradation RhB under visible-light irradiation was fitted in Fig. 8b, and the degradation kinetics of RhB was described by the first-order kinetics curves. Degradation experiments followed the first-order kinetic equation ln(C_0_-C) = − K*t* + b (in which *K* is the apparent first-order rate constant and *t* is the reaction time). By comparing the rate constants (*K* values) of ZnTCP-sensitized TiO_2_-HNBs catalysts, we can see that the rate constants of these catalysts increase first and then decrease with the ZnTCP/TiO_2_-HNBs mass ratio increase. Observed from the rate constants histogram (Fig. 8b), we can find that T-2p reveals the highest visible-light photocatalytic activity (rate constant of 0.7139), which is 3.6 times higher than that of TiO_2_-HNBs (T-0p) (rate constant of 0.19841); furthermore, the photocatalytic rate constants of other ZnTCP@TiO_2_-HNBs samples are basically larger than that of TiO_2_-HNBs.

The possible reasons is that may be because ZnTCP-sensitized TiO_2_-HNBs can extend the absorption wavelength range of TiO_2_-based catalysts, reducing the bandgap of TiO_2_, and facilitate the effective separation of the electron-hole pairs generated by TiO_2_-HNBs after absorbing visible light, thereby improving the utilization of electron-hole pairs. However, too much ZnTCP-sensitized parts may result in the increase of defects on the surface of TiO_2_-based catalyst, resulting in a rapid recombination center of photogenerated electrons and holes, thereby decreasing the photocatalytic activity.

In order to investigate the long-term stability of as-prepared ZnTCP@TiO_2_-HNBs, the photocatalysts were recycled after each photocatalytic degradation experiment and then were reused in the next run after washing treatment. Figure 8c shows sample T-2p were recycled to 5 rounds after bleaching RhB under visible-light irradiation; as the number of photodegradation cycles increased, the photocatalytic rate of RhB still maintained a high catalytic activity, indicating that the active site of ZnTCP@TiO_2_-HNBs surface did not decrease, and the composite photocatalyst has good photocatalytic stability under visible-light irradiation. The strong bridging ester bond-linking leads to a slower decrease of the degradation efficiency for sample T-2p. These results further demonstrate that ZnTCP can establish a steady chemical bridging bond-linking interaction between ZnTCP and TiO_2_-HNBs, thus enhancing the visible-light photocatalytic performance, stability, and recyclability of ZnTCP@TiO_2_-HNBs. XRD shows that the T-2p is still anatase type and did not change the crystal type after many cycles, further verifying the stability of the catalyst (Fig. 8d).

### PL Analysis

**Fig. 9:**
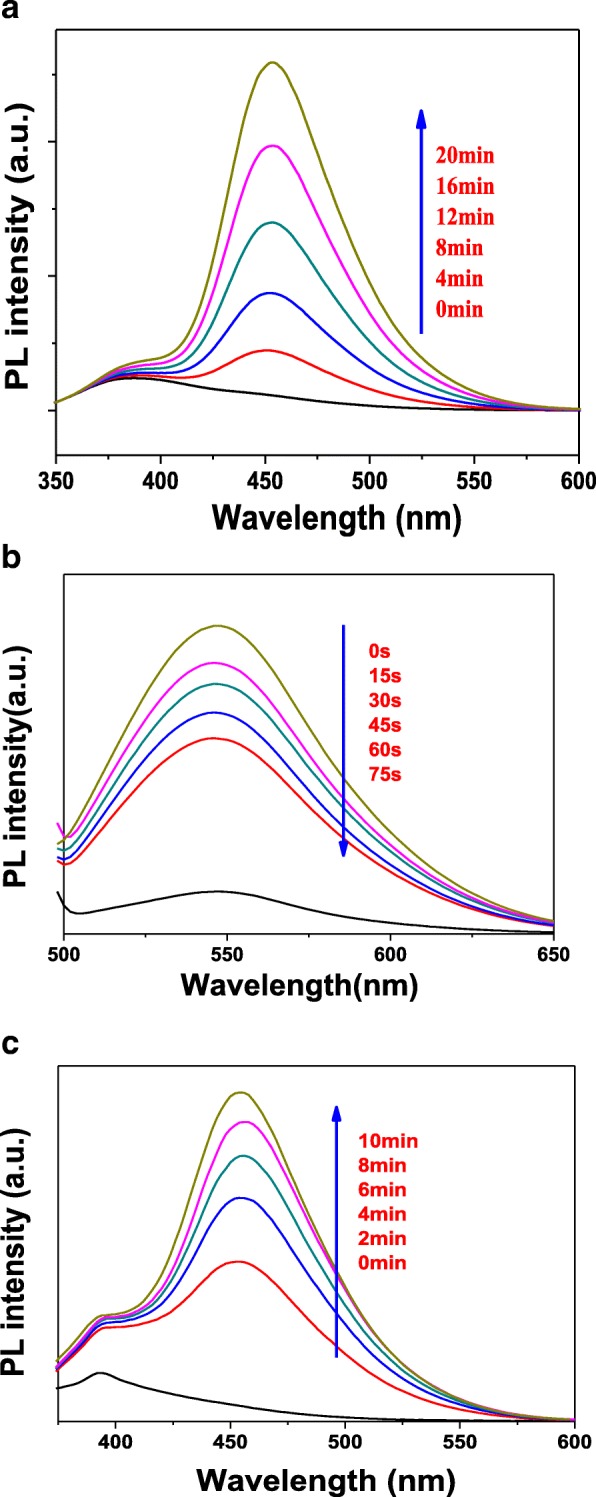
Fluorescent detection of active species •OH (**a**), •O_2_^−^ (**b**), and ^1^O_2_ (**c**) with T-2p as photocatalyst

In order to study the active species generated during photocatalytic degradation, the coumarin, 4-chloro-7-nitrobenzo-2-oxa-1,3-diazole (NBD-Cl), and 1,3-diphenylisobenzofuran (DPBF) were used as the hydroxyl radical (•OH), superoxide radical (•O_2_^−^), and singlet oxygen (^1^O_2_) fluorescent probe molecules, respectively. Figure 9a displays the active species of hydroxyl radical detection map with T-2p as photocatalyst. As shown in Fig. 9a, the fluorescence emission wavelength was centered at 450 nm and the fluorescence intensity gradually increased, indicating the concentration of •OH group was increasing with the increase of illumination time. The reason is due to the fact that the continuously generated •OH active group and coumarin react to obtain 7-hydroxycoumarin, the amount of the reaction is gradually increased, and the fluorescence intensity is gradually increased [44, 45].

It is well accepted that quantification of superoxide ion concentration can be monitored its reaction with 4-chloro-7-nitrobenzo-2-oxa-1,3-diazole (NBD-Cl) by measuring its fluorescence emission intensity at 550 nm using an excitation wavelength of 470 nm. According to Fig. 9b, it can be found that the fluorescence emission wavelength was centered 550 nm and the fluorescence intensity gradually decreased with the increase of illumination time. In the presence of NBD-Cl, the degradation rate is not obviously increased. The results indicate that •OH radical is hardly generated, while •O_2_^−^ is the significant active species generated in the system. More e^−^ can react with O_2_ to form superoxide radical •O_2_^−^ instead of the rapid recombination with h^+^, which further improves the photodegradation activity and testifies the catalyst can generate the active species •O_2_^−^.

In addition, a typical fluorescence method for detecting singlet oxygen (^1^O_2_,) by using 1,3-diphenylisobenzofuran (DPBF) as a probe molecule is proposed, which exhibited a strong fluorescence spectrum with a maximum at 455 nm when excited at 410 nm. As shown in Fig. 9c, the fluorescence emission wavelength was centered 455 nm and the fluorescence intensity gradually increased with the increase of illumination time. Based on the above observation, it can be concluded that the photocatalyst produces a certain amount of singlet oxygen in the light condition. To sum up, the ZnTCP@TiO_2_-HNBs catalyst can generate •OH, •O_2_^−^, and ^1^O_2_ active species during the photodegradation process under visible-light irradiation.

### Electrochemical Analysis

**Fig. 10:**
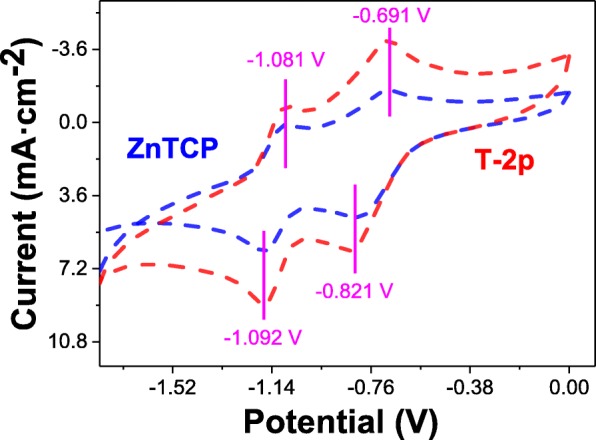
Cyclic voltammetry analysis of 1.0 × 10^−3^ mol dm^−3^ of ZnTCP and T-2p on Pt in 0.1 mol dm^−3^ TBAP/DMF vs. SCE. Scan rate: 0.1 V s^−1^

To attain more insights into photocatalytic transformation for oxidation degradation organic pollutants, the electrochemical properties of ZnTCP and ZnTCP@TiO_2_-HNBs (T-2p) in DMF were investigated with cyclic voltammetry (CV) on a glassy carbon electrode using tetrabutylammonium perchlorate as supporting electrolyte. It can be clearly seen that two complete oxidation and reductions processes for ZnTCP observed from the potential window are shown with blue dashed lines in Fig. 10. Furthermore, ZnTCP@TiO_2_-HNBs (T-2p) distinctly displayed similar two oxidation processes resulted from the ZnTCP center with red dashed lines [39]. The oxidation-reduction electrochemical behavior of ZnTCP complexes mainly occurred on the porphyrins ring and did not involve the change in the valence state of the central metal ions, which were good agreement with our previous reported [47]. However, the oxidation degradation reaction of ZnTCP@TiO_2_-HNBs (T-2p) was very similar as that of ZnTCP, which indicated the photocatalytic transformation mainly happened on the porphyrin ring, not relative to the center zinc ion and TiO_2_-HNBs. These results further confirm that the strong interaction, such as conjugated ester chemical bonds between TiO_2_-HNBs and ZnTCP, was established rather than simple physical adsorption interaction.

### Proposed Probable Photocatalytic Mechanism

**Fig. 11:**
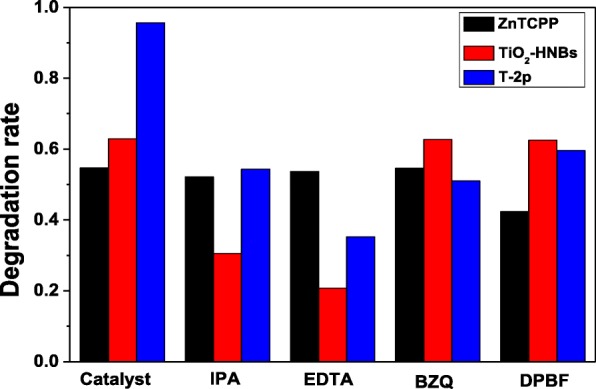
The C _t_/C_0_ vs. time curves of different reactive species scavengers on the photodegradation of RhB under visible-light irradiation

Much effort has been devoted to illustrate the photocatalytic mechanism for oxidation degradation organic pollutants. In order to speculate the photodegradation mechanism, the main oxidative species in the photocatalytic process was firstly detected through radical and hole trapping experiments by using EDTA (hole h^+^ scavenger), *p*-benzoquinone (BZQ, superoxide radical •O_2_^−^ scavenger), *iso*-propyl alcohol (IPA, hydroxyl radical •OH scavenger) and 1,3-diphenylisobenzofuran (DPBF, singlet oxygen ^1^O_2_ scavenger). Figure 11 shows the Ct/C0 vs. time curves of different reactive species scavengers on the photodegradation of RhB under visible-light irradiation.

As displayed in Fig. 11, the photocatalytic degradation of RhB was apparently restrained after the injection of the active species scavenger. Taking the decreasing photodegradation rate into consideration, it can be found that RhB degradation rate with ZnTCP as a photocatalyst mainly depended on active species of ^1^O_2_ group according to the 1,3-diphenylisobenzofuran (DPBF) detection results. Furthermore, RhB photodegradation rate with TiO_2_-HNBs as catalysts mostly depended on active species of h^+^ and •OH group. However, it is very clear that RhB photodegradation rate with ZnTCP@TiO_2_-HNBs (T-2p) as catalysts was influenced by all active species of hole (h^+^), hydroxyl radical (•OH), superoxide radical (•O_2_^−^), and singlet oxygen (^1^O_2_), further confirming ZnTCP@TiO_2_-HNBs (T-2p) can generate these active species during the photodegradation process.

**Fig. 12:**
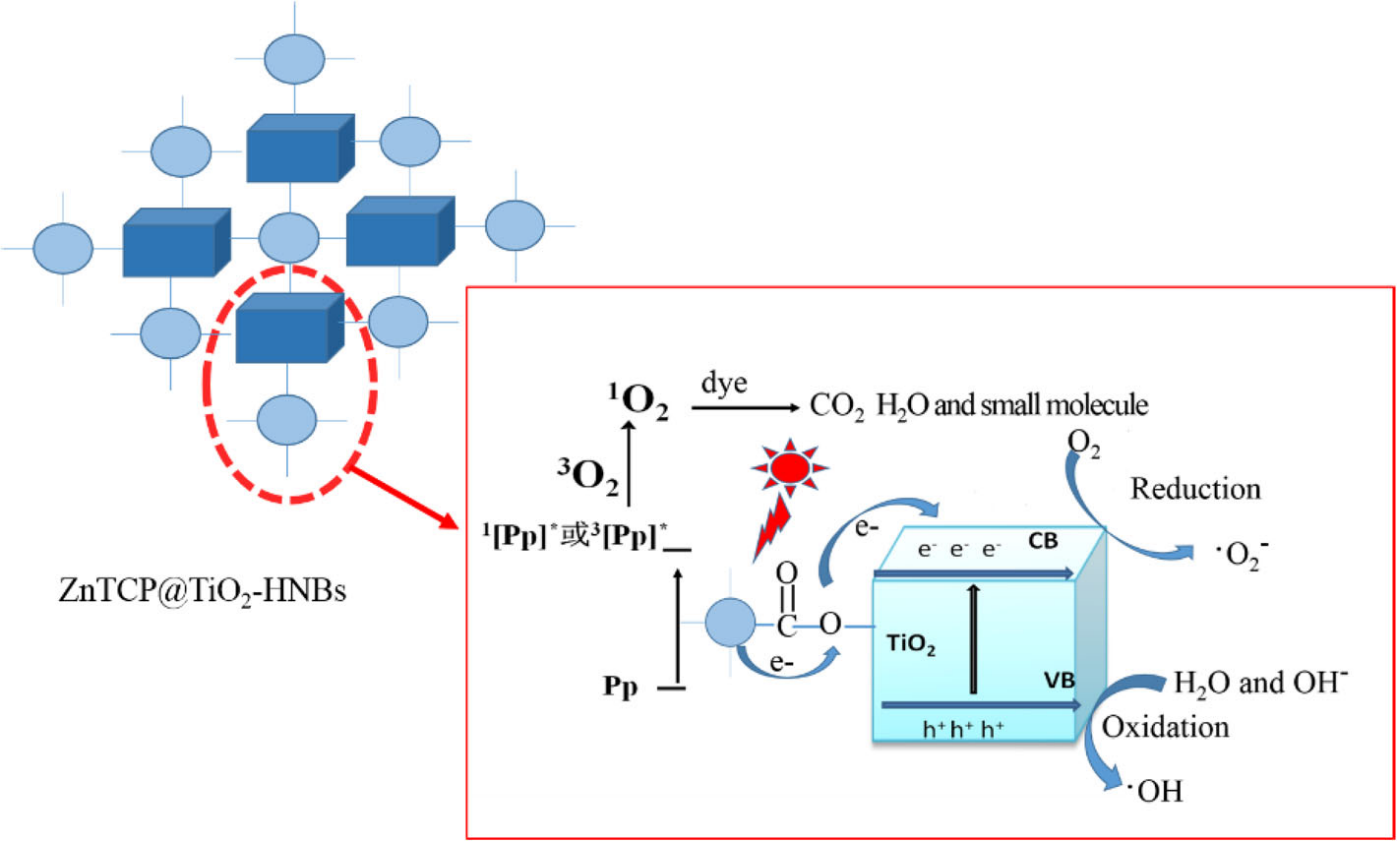
Proposed probable photocatalytic mechanism of ZnTCP@TiO_2_-HNBs

The probable photodegradation mechanism of organic dyes with ZnTCP-sensitized TiO_2_-HNBs as catalysts under visible-light irradiation is shown in Fig. 12. The degradation process generally consists of three moieties. The first part mainly involves the generation of singlet oxygen (^1^O_2_). When irradiated by simulated sunlight, the inspired electrons of ZnTCP are transferred from the ground state of porphyrin [Pp] to the excited singlet state ^1^[Pp]* [47], and then, relaxation of the singlet excited state generates the triplet excited state ^3^[Pp]* through a process of intersystem crossing. Electrons from ^1^[Pp]* and ^3^[Pp]* excited states are trapped by the adsorbed O_2_ to form singlet oxygen (^1^O_2_), which can cause RhB degradation into pieces of small organic molecule or mineralization to CO_2_.

In contract, superoxide radical (•O_2_^−^) can be generated in the second moiety. Biomimetic catalytic moiety of ZnTCP is not only responsible to generate singlet oxygen (^1^O_2_), but also acts as electron bridges from ^1^[Pp]* and ^3^[Pp]* excited states transferred to conduction band (CB) of TiO_2_-HNBs, which can be trapped further by the adsorbed O_2_, resulting in formation of an amount of •O_2_^−^ causing photodegradation of RhB present on the surface of TiO_2_-HNBs.

On the other hand, in the sensitization of porphyrins, the electrons of TiO_2_ are excited from valence band (VB) to conduction band (CB) forming electron-hole pairs (e^−^-h^+^) during the visible-light irradiation [48, 49]. The photogenerated holes transfer to the surface of TiO_2_ reacted with H_2_O or OH^−^, resulting in formation of hydroxyl radical (•OH). The photogenerated electrons are transferred to the surface of TiO_2_ trapped by the adsorbed oxygen molecule, obtained an amount of superoxide radical (•O_2_^−^), and these active species (•O_2_^−^, •OH) mainly implement the degradation of organic dyes by its successive formation of intermediaries participated.

Thus, overall, a cooperative mechanism is proposed for the degradation of organic dyes involving three components of photocatalytic system. The enhanced visible-light photodegradation activities of Zn(II)TCP@TiO_2_-HNBs compared to ZnTCP and TiO_2_-HNBs might be related to synergetic generation of three active species (•O_2_^−^, •OH, and ^1^O_2_), resulting in more higher efficient photocatalytic activities.

## Conclusions

A facile one-step solvothermal treatment via a topotactic transformation process was employed to synthesize a series of visible-light-driven biomimetic photocatalysts based on ZnTCP-sensitized TiO_2_ hollow nanoboxes assembled by six ordered nanosheets with high-energy {001} facets dominant exposure. ZnTCP played an important role in formation ester bonds to construct 3D hollow nanoboxes and transfer photogenerated electrons to sensitize TiO_2_-HNBs for enhancing visible-light response. Due to synergistic visible photodegradation mechanism of biomimetic catalyst, which can produce not only hydroxyl radical (•OH) and hyperoxygen radical (•O_2_^−^) from TiO_2_, but also singlet oxygen (^1^O_2_) generated by biomimetic enzyme porphyrin, the photocatalytic degradation RhB rate constants of ZnTCP@TiO_2_-HNBs (T-2p) were greatly enhanced with a degradation yield of 99%, much larger (3.6 time) than TiO_2_-HNBs under visible-light irradiation. This novel approach is expected to provide a perfect reference for perfect dye-sensitized method to synergistically fabricate other surface modification TiO_2_-based composites, which is of great value with promising application for purifying domestic sewage.

## Abbreviations

3D: Three-dimensional
CV: Cyclic voltammetry
DRS: UV-vis diffuse reflectance spectroscopy
IR: Infrared spectra
PL: Photoluminescence
SEM: Scanning electron microscope
TEM: Transmission electron microscope
TiO_2_-HNBs: TiO_2_ hollow nanoboxes
UV-vis: UV-vis spectroscopy
XPS: X-ray photoelectron spectroscopy
XRD: X-ray diffraction
ZnTCP: Zinc(II) *meso*-tetra(4-carboxyphenyl)porphyrinato
ZnTCP@TiO_2_-HNBs: Zinc(II) *meso*-tetra(4-carboxyphenyl)porphyrinato-sensitized TiO_2_ hollow nanoboxes
